# One Year Clinical Evaluation of a Low Shrinkage Composite Compared with a Packable Composite Resin: A Randomized Clinical Trial

**Published:** 2017-03

**Authors:** Razieh Hoseinifar, Elaheh Mortazavi-Lahijani, Hassan Mollahassani, Ahmad Ghaderi

**Affiliations:** 1 Assistant Professor, Oral and Dental Diseases Research Center, Kerman, Iran; Kerman Social Determinants on Oral Health Research Center Kerman, Iran; Department of Operative Dentistry, School of Dentistry, Kerman University of Medical Sciences, Kerman, Iran; 2 Postgraduate Student, Department of Restorative Dentistry, School of Dentistry, Kerman University of Medical Sciences, Kerman, Iran; 3 Instructor, Department of Operative Dentistry, School of Dentistry, Zahedan University of Medical Sciences, Zahedan, Iran

**Keywords:** Composite Resins, Dental Marginal Adaptation, Patients

## Abstract

**Objectives:**

The aim of this study was to evaluate the clinical performance of a packable and a low shrinkage methacrylate-based composite after one year.

**Materials and Methods:**

In this clinical trial, 50 class I or II restorations were placed in 25 patients. Each patient received two restorations. The tested materials were: (I) Filtek P60 + Single Bond 2 and (II) Kalore GC + Single Bond 2. The restorations were evaluated by two independent examiners after one week (baseline), six months and one year according to the modified United States Public Health Service (USPHS) criteria. The evaluated parameters included color match, marginal adaptation, anatomical form, retention, surface texture, postoperative sensitivity, marginal staining and secondary caries. Data were then analyzed using Friedman and conditional (matched) logistic regression tests at P<0.05 level of significance.

**Results:**

P60 and Kalore performed similarly at six months and one year (P>0.05). When each composite resin was evaluated independently at baseline and after one year, no statistically significant differences were found except for marginal adaptation (P60) where four restorations were rated Bravo (clinically acceptable). In 8% of restorations, patients expressed postoperative sensitivity.

**Conclusions:**

Kalore GC and Filtek P60 showed acceptance clinical performance after one year of service.

## INTRODUCTION

The improvements in dental adhesives and composite resins, along with increased demand for tooth-colored restorations in addition to a minimally invasive approach for caries treatment, have made composites the primary choice for direct posterior restorations in many countries [1,2]. Recent clinical studies indicated acceptable clinical performance of composite restorations and some even provided 10- to 20-year outcomes showing low annual failure rates of approximately 2% [3,4]. However, the polymerization shrinkage seems to be the major limitation of composite resins in posterior restorations [5].

This volumetric shrinkage ranges from 2 to 5% which may cause debonding at the tooth-composite interface and subsequently lead to postoperative sensitivity, marginal staining, recurrent caries and microleakage. Application of low-shrinkage composites is a key strategy to reduce polymerization shrinkage stress [6,7]. Two main approaches to reduce polymerization shrinkage include using different types of resin monomers and reduction of reactive sites per volume unit (by increasing filler loading and/or increasing molecular weight per reactive group) [8].

Kalore (GC Corporation, Tokyo, Japan) is a low-shrinkage nano-composite with 82% filler loading (by weight), that is based on DuPont technology. The DuPont monomer (DX-511) is a high molecular weight urethane dimethacrylate (UDMA) monomer with a low number of C=C double bonds. The low polymerization shrinkage of Kalore (1.7%) is due to the high molecular weight of DX511 and the presence of low number of C=C double bonds [9].

In order to overcome difficulties in composite placement and establishment of appropriate proximal contact, packable composite resins with changes in handling properties were introduced to the dental market in the late 1990s. These composites are stiffer and less sticky than conventional composites, which allows for their easier placement [10,11]. Manufactures have eliminated composite stickiness by altering the filler morphology (Surefil, ALERT and Solitaire) or the resin matrix monomers (Prodigy and Filtek P60) [11]. The oral conditions such as thermal changes, presence of microorganisms, saliva, oral hygiene and masticatory stresses can interfere with the durability of posterior composite restorations by increasing the surface roughness, marginal microleakage and secondary caries [12] and the clinical trials are the final test to measure the durability and clinical effectiveness of adhesives and dental composites [13]. Thus, the aim of this clinical trial was to evaluate the clinical performance of a packable composite in class I and II restorations compared with a low-shrinkage methacrylate-based composite after one year of service.

## MATERIALS AND METHODS

The study protocol was approved by the Ethics Committee of Kerman University of Medical Sciences (k.A/92/480). This study was also registered in the Iranian Registry of Clinical Trials with registration code IRCT2016070528804N1. In this randomized clinical trial, a total of 50 restorations were placed in 25 patients (age range of 18–40 years) in the Department of Operative Dentistry of Kerman University of Medical Sciences. Each patient received two class I or II restorations. Half of the prepared cavities were randomly restored with A2 shade of P60 composite (3M, ESPE, St. Paul, MN, USA) and the other half with A2 shade of Kalore GC composite (GC Corporation, Tokyo, Japan) (Table 1). Before participating in the study, patients were properly informed about the study and informed consent was obtained from each patient.

**Table 1:** Number of restorations by location (tooth) and number of surfaces for each restorative system

**Restorative system**	**Number of restorations**	**Premolar**	**Molar**	**Class I**	**Class II (MO or DO)**
**Filtek P60 + Adper Single Bond 2**	25	5	20	19	6
**Kalore-GC + Adper Single Bond 2**	25	4	21	19	6
**Total (%)**	50 (100)	9 (18)	41 (82)	38 (76)	12 (24)

Patients with poor oral hygiene, parafunctional habits, serious health problems, a known allergy to the substances used in this study, history of existing tooth hyper-sensitivity, pregnancy or breast-feeding, chronic use of anti-inflammatory, analgesic, and psychotropic drugs, xerostomia and periodontal diseases were excluded. Bitewing radiographs of the teeth to be restored were taken preoperatively.

### Clinical procedure:

The teeth to be restored were cleaned with a slurry of pumice, rinsed with water and then air dried. The conservative cavity was prepared using a diamond bur (Tizcavan, Tehran, Iran) with a high-speed hand-piece. New burs were used after every five preparations. All carious tooth structures were removed using a low-speed hand-piece and a steel round bur. In very deep cavities (less than approximately 0.5mm of dentin) the dentin was covered with calcium hydroxide (Dycal, Dentsply, Caulk, Milford, USA) and resin modified glass-ionomer cement (GC Corporation, Tokyo, Japan); whereas, in deep cavities (the residual dentin thickness was 0.5–1.5mm) dentin was covered solely with resin-modified glass-ionomer cement.

Stainless steel matrix band (Hahnenkratt, Königsbach-Stein, Germany) and Tofflemire matrix retainer (Water Pik, Inc., Fort Collins, CO, USA) with wooden wedges were used for class II cavities. Enamel and dentin surfaces were etched with 35% phosphoric acid (TotalEtch, Ivoclar Vivadent, Schaan, Liechtenstein) for 15 seconds, rinsed with water for 10 seconds and dried with cotton pellet, leaving a moist surface. Then Single Bond 2 (3M ESPE, St. Paul, MN, USA) adhesive system was applied to the etched surfaces in accordance with the manufacturer’s instructions, and then light cured for 20 seconds at 800mW/cm^2^ intensity using a LED curing unit (Demi Plus; Kerr, Orange, CA, USA), and checked periodically with a radiometer (Demetron LED Radiometer; Kerr, Orange, CA, USA). The teeth to be restored were randomly filled with a packable composite (P60), or a low-shrinkage methacrylate-based composite (Kalore GC). The resin composites were placed in straight increments not exceeding 2mm in thickness, and adapted with a flat-faced condenser and then each increment was light cured for 40 seconds.

The materials used in this study with their chemical compositions are presented in Table 2.

**Table 2:** Description of the bonding system and composite resins used in this study

**Material**	**Composition**	**Filler loading (wt)**	**Manufacturer**
Filtek P60 **(N548204)**	Bis-GMA, UDMA, Bis-EMA Zirconia/silica, DX-511, UDMA, dimethacrylate co-monomers, Prepolymerized filler (with lanthanoid fluoride), fluoro-alumino-silicate glass, strontium/barium glass, silicon dioxide, lanthanoid fluoride	80%	3M ESPE, St. Paul, MN, USA
GC Kalore **(1306101)**		82%	GC Corporation, Tokyo, Japan
Adper Single Bond 2 **(N567778)**	BisGMA, HEMA, dimethacrylate, polyalkenoic, acid copolymer, initiator, ethanol and water		3M ESPE, St. Paul, MN, USA

All restorations were finished with fine grit finishing diamond burs (Diatech; Dental AG, Heerbrug, Switzerland) and polished with polishing rubber points (Edenta Composite Polishing Kit; AU, St. Gallen, Switzerland) and Enhance (Dentsply Latin America, Petropolis, RI, Brazil).

### Clinical evaluation:

All restorations were evaluated one week after placement, six months and one year. Two calibrated clinicians, other than the operator, evaluated the restorations blindly at each recall using the modified United States Public Health Service (USPHS) criteria (Table 3). The two examiners, patients and analyzer were unaware of the type of composite used (triple-blind design). The evaluated parameters were color match, marginal adaptation, retention, anatomical form, sensitivity, surface roughness, marginal staining and secondary caries. For each criterion, Alpha score represented ideal clinical situation, Bravo indicated clinically acceptable, and Charlie indicated clinically unacceptable situation.

**Table 3:** Modified USPHS criteria used for clinical evaluation

**Criteria**	**Code**	**Definition**
**Color match**	Alpha	Restoration matches adjacent tooth structure in color and translucency.
	Bravo	Mismatch is within an acceptable range of tooth color and translucency
	Charlie	Mismatch is outside the acceptable range.

**Marginal adaptation**	Alpha	Restoration closely adapted to the tooth. No crevice visible. No explorer catch at the margins, or there was a catch in one direction.
	Bravo	Explorer catches. No visible evidence of a crevice into which the explorer could penetrate. No dentin or base visible
	Charlie	Explorer penetrates into a crevice that is of a depth that exposes dentin or base.

**Surface roughness**	Alpha	Surface of restoration is smooth.
	Bravo	Surface of restoration is slightly rough or pitted, but can be refinished.
	Charlie	Surface deeply pitted, irregular grooves, and cannot be refinished.

**Sensitivity**	Alpha	None.
	Bravo	Mild but bearable.
	Charlie	Uncomfortable, but no replacement is necessary.

**Anatomical form**	Alpha	Restorations continuous with existing anatomic form.
	Bravo	Restorations discontinuous with existing anatomic form but missing material not sufficient to expose dentin base.
	Charlie	Sufficient material lost to expose dentin or base.

**Retention**	Alpha	Full retention.
	Bravo	Partial retention.
	Charlie	Restoration is lost.

**Marginal staining**	Alpha	No staining along cavosurface margin.
	Bravo	<50% of cavosurface affected by stain (removable, usually localized).
	Charlie	>50% of cavosurface affected by stain.

**Secondary caries**	Alpha	Absent.
	Bravo	Present.

When disagreements occurred during the evaluation of restorations, a consensus was reached between the two examiners. Data were analyzed using SPSS version 18.0 (SPSS Inc., IL, USA) and the Friedman and conditional (matched) logistic regression tests at P<0.05 level of significance.

## RESULTS

Overall in 25 patients, fifty restorations were placed and all restorations were evaluated at six months and one-year follow-up. The results are summarized in Table 4. The results of the present study indicated that all of the restorations at baseline exhibited Alpha score for all criteria (except for postoperative hypersensitivity and color match). Four patients experienced postoperative hypersensitivity (slight discomfort associated with cold beverage and mastication) after restoration placement (two P60 and two Kalore restorations), which disappeared gradually after three months.

**Table 4:** Clinical rating of restorations at baseline, six months and one year

**Criteria**	**Code**	**One week (Base)**		**Six months**		**One year**	

		**P60**	**Kalore**	**P60**	**Kalore**	**P60**	**Kalore**
**Color match**	A	21	22	21	22	21	22
	B	4	3	4	3	4	3
	C	-	-	-	-	-	-

**Marginal adaptation**	A	25	25	23	24	21	23
	B	-	-	2	1	4	2
	C	-	-	-	-	-	-

**Surface roughness**	A	25	25	24	25	22	24
	B	-	-	1	-	3	1
	C	-	-	-	-	-	-

**Sensitivity**	A	23	23	25	25	25	25
	B	2	2	-	-	-	-
	C	-	-	-	-	-	-

**Anatomical form**	A	25	25	25	25	25	25
	B	-	-	-	-	-	-
	C	-	-	-	-	-	-

**Retention**	A	25	25	25	25	25	25
	B	-	-	-	-	-	-
	C	-	-	-	-	-	-

**Marginal staining**	A	25	25	25	25	25	24
	B	-	-	-	-	-	1
	C	-	-	-	-	-	-

**Secondary caries**	A	25	25	25	25	25	25
	B	-	-	-	-	-	-
	C	-	-	-	-	-	-

No statistically significant differences were found between the two composites at six months and one year of service (P>0.05). Moreover, when each composite was evaluated independently at baseline and after one year, no statistically significant differences were found (P>0.05) except in P60 group for marginal adaptation (P<0.05). No secondary caries, lack of retention and loss of anatomical form were found in any of the studied materials after one year (100% Alpha).

At the six-month recall, 6% of restorations (2% Kalore and 4% P60) showed Bravo score for marginal adaptation and 2% (P60) for surface roughness. After one year of follow-up, following restorations were recorded as Bravo: 8% surface roughness (2% Kalore and 6% P60), 12% marginal adaptation (4% Kalore and 8% P60) and 2% marginal staining (P60).

## DISCUSSION

The result of this study showed that when each composite resin was evaluated independently at baseline and after one year, no statistically significant differences were found except in P60 group that showed significant difference in marginal adaptation. This finding was in accord with the results of previous studies, which have reported similar performance of P60 composite [14,15].

Kiremitci et al, [13] in 2009 evaluated six years of clinical performance of Filtek P60 composite, and demonstrated the acceptable clinical performance of P60 after six years of service. In their study, all Filtek P60 restorations showed Alpha ratings in all of the evaluated USPHS criteria after one year.

Loguercio et al, [11] in 2006 evaluated three-year clinical performance of four packable composites and one hybrid composite. They reported that Filtek P60 exhibited excellent clinical performance after one year. They also observed an excellent color match of P60 after three years of follow-up and reported that this excellent color match is due to the high percentage of UDMA in the organic matrix, which is less prone to water sorption than bisphenol A-glycidyl methacrylate (Bis-GMA)-based composite. In the current study, after one week, 14% of restorations represented Bravo score for color match, but they remained unchanged over the one-year observation period.

Gianordoli Neto and others [14] in 2008 evaluated one-year clinical performance of two composites (Filtek Z250 and Filtek P60) and found no significant differences between the two after one year. They also reported that 91.4% of restorations showed Alpha score for marginal adaptation at one-year recall. Yazici et al, [15] in 2014 compared the three-year clinical performance of Filtek P60 with Filtek P90 and reported no significant difference between the two composites after three years of evaluation. Moreover, 97% of P60 restorations showed Alpha score for surface roughness and marginal adaptation at one-year follow-up.

The clinical performance of Kalore restorations after one year showed also minor changes compared to baseline. Kalore is a nano-composite that is based on DuPont technology. The DuPont molecule (DX-511) is a high molecular weight UDMA monomer with a low number of C=C double bonds. The molecular weight of this monomer is twice that of Bis-GMA or UDMA. DX-511 monomer has a long rigid molecular core and flexible arms in the structure. The long rigid core prevents the deformation of monomer and decreases polymerization shrinkage. This monomer is compatible with the current bonding systems and composites. The low polymerization shrinkage of Kalore (1.7%) is due to the high molecular weight of DX511 and presence of low number of C=C double bonds [9].

Based on our results, 8% of restorations represented postoperative sensitivity at one week, while no hypersensitivity was detected after six months. There is substantial evidence that the rate of postoperative hypersensitivity varies greatly among reported data. Several clinical studies indicated that 30% of patients had postoperative sensitivity after posterior composite restorations [16, 17]. Opdam et al, [18] in 1998 reported that 14% prevalence of postoperative hypersensitivity after placement of class I composite restorations. Briso and colleagues [16] in 2007 assessed 143 class I and II composite restorations and detected 5% postoperative hypersensitivity in class I and 15% in class II restorations, while Loguercio et al, [11] in 2006 and Kiremitci et al, [13] in 2009 evaluated the clinical performance of Filtek P60 restorations, and observed no postoperative hypersensitivity in P60 restorations.

The postoperative hypersensitivity in posterior composite restorations is reported as a common problem in operative dentistry. Postoperative hypersensitivity may be due to several factors such as etching of dentin, bacterial microleakage, cuspal flexure, cavity depth, technique of restoration placement, over-drying of dentin, occlusal interferences, incorrect adhesive procedure, cavity size and trauma caused by cavity preparation [19,20]. Variations in the occurrence of postoperative sensitivity have also been reported among different clinicians with respect to their techniques and experiences [19].

Our results indicated that marginal adaption in 12% of restorations (8% Filtek P60 and 4% Kalore) rated Bravo after one year of follow-up and no significant difference was found between the two experimented composites.

The marginal adaptation of composite restoration is influenced by several factors such as type of dentin adhesive, the polymerization shrinkage of composites, restoration technique and accuracy of the finishing procedure [21,22]. Some studies have reported that many of marginal defects result from the fracture of thin areas of composite flash or excessive adhesive and these external flashes can be removed by better finishing and polishing [12,22]. The results of our study exhibited that after one year, 8% of restorations (2% Kalore and 6% P60) showed Bravo score for surface roughness. Consequently, Kalore restorations showed relatively better performance in comparison with Filtek P60, although no significant difference was recorded between the two types of composites. However, marginal adaption of P60 was significantly worse (but clinically acceptable) at one year than at baseline. The surface roughness of composite restorations depends on some internal factors such as filler (type, size, shape, hardness and loading), type of resin matrix, ultimate degree of conversion and quality of bond between filler particles and resin matrix [23]. The external factors include the techniques of finishing and polishing, type of finishing and polishing used and light curing method [24].

In the current study, Filtek P60 expressed relatively higher surface roughness; it could be related to the deficiency of bonding between the matrix and the fillers resulted from non-silanization of the latter. This may cause protrusion of some filler particles as the weak resin matrix is lost during the procedure of finishing and polishing, and as the result, the surface becomes rough [25]. Moreover, Filtek P60 contains high molecular weight Bis-EMA and UDMA resulting in a slightly softer matrix because of forming fewer double bonds and thereby increasing the surface roughness [24].

On the other hand, some experiments have indicated that microhybrid composites in comparison with nano-filled composites show smaller volume and less homogenous distribution of inorganic fillers. A surface formed by nanoparticles usually shows less particle loss (and thus increased surface roughness), as compared with microhybrid composites [26].

In the current study, no failure was detected among restorations during the one-year period. Collin et al, [27] in 1998 reported that recurrent caries and bulk fracture are the main factors responsible for posterior composite failure and in their study, secondary caries and bulk fracture were not observed. Clinical success of posterior composite restorations depends on several factors including patient caries risk, oral hygiene and age, clinical factors (size, location and type of restoration), socioeconomic factors and operator-related factors (knowledge, skills, quality of work and technique) [28].

Although one or two-year studies may provide useful information on the performance of newly introduced composites and their catastrophic failure, precise assessment of longevity of composite resins, however, requires long-term evaluation.

## CONCLUSION

Kalore GC and Filtek P60 showed equally acceptable clinical performance after one year of service.
